# NFATc1 regulates LAG3^*+*^CD8^*+*^ T cells in the spleen of mice infected with *Plasmodium yoelii* NSM

**DOI:** 10.1371/journal.pntd.0013605

**Published:** 2025-10-06

**Authors:** Xingfei Pan, Feng Mo, Li Pan, Wei Xiao, Guikuan Liang, Xiongyu Xie, Haiwen Yuan, Haixia Wei, Shan Zhao, Lu Li, Lei Jia, Hongyan Xie, Jun Huang

**Affiliations:** 1 Department of Infectious Diseases, Guangdong Provincial Key Laboratory of Major Obstetric Diseases, Guangdong Provincial Clinical Research Center for Obstetrics and Gynecology, The Third Affiliated Hospital of Guangzhou Medical University, Guangzhou, China; 2 Key Laboratory of Immunology, Sino-French Hoffman Institute, Guangzhou Medical University, Guangzhou, China; 3 Guangxi Key Laboratory of Intelligent Precision Medicine, Nanning, China; 4 International Health Medicine Innovation Center, Shenzhen University, Shenzhen, China; 5 The Second Affiliated Hospital, Guangdong Provincial Key Laboratory of Allergy & Clinical Immunology, The State Key Laboratory of Respiratory Disease, Guangzhou Medical University, Guangzhou, China; 6 The Sixth Affiliated Hospital of Guangzhou Medical University, Qingyuan People’s Hospital, Qingyuan, China; University of Liverpool, UNITED KINGDOM OF GREAT BRITAIN AND NORTHERN IRELAND

## Abstract

Malaria, an infectious disease caused by Plasmodium, is primarily characterized by anemia and splenomegaly. CD8 ⁺ T cells are known to play a key role in anti-malaria immunity. Lymphocyte Activation Gene 3 (LAG3), a critical immune checkpoint molecule, is pivotal in CD8 ⁺ T cell-mediated anti-tumor responses. However, the role of LAG3 ⁺ CD8 ⁺ T cells in anti-malarial immunity and the regulatory factors governing LAG3 expression in CD8 ⁺ T cells remain unclear. In this study, C57BL/6 mice were subcutaneously infected with *Plasmodium yoelii* NSM. Splenic lymphocytes were isolated and analyzed using flow cytometry (FACs) and single-cell RNA sequencing (scRNA-seq). Results showed a significant upregulation of LAG3 expression in splenic CD8 ⁺ T cells post-infection. These LAG3 ⁺ CD8 ⁺ T cells displayed enhanced activation, responsiveness, proliferative capacity, and cytokine production. Additionally, activated nuclear factor of activated T cells 1 (NFATc1) was found to co-express with LAG3 in splenic CD8 ⁺ T cells from infected mice. Dual-fluorescence reporter gene assays in 293T cells identified NFATc1 as a key transcription factor that binds to the LAG3 promoter sequence. Knockdown of NFATc1 *via* small interfering RNA (siRNA) reduced LAG3 expression. In conclusion, our findings suggest that splenic LAG3 ⁺ CD8 ⁺ T cells in *Plasmodium yoelii* NSM-infected C57BL/6 mice display enhanced functionality and imply that NFATc1 could positively regulate LAG3 expression.

## Author Summary

The authors observed that in the spleens of mice infected with malaria parasites, the amount of Lag3 molecules on CD8 + T cells (a type of immune cell) increased significantly. What’s more, these CD8 + T cells that had Lag3 molecules were highly active. They could multiply well and were good at releasing many different kinds of cytokines. The authors believe Lag3 is probably a molecule that only starts to appear after T cells are activated.At the same time, the authors also found that NFATc1 might be an important factor that helps trigger the production of Lag3 molecules. They learned this by doing experiments at the cellular and molecular levels, such as single-cell RNA sequencing and flow cytometry (common lab techniques). When they used a method called RNA interference to lower the amount of NFATc1, they found that the production of Lag3 molecules was also reduced. The authors think NFATc1 could become a potential “target gene” meaning in the future, it might be used to stop Lag3 molecules from forming, which could help treat diseases.

## Background

Malaria is an infectious disease caused by *Plasmodium*. Human-infecting species include *Plasmodium falciparum*, *P. vivax*, *P. malariae*, *P. ovale*, and *P. knowlesi* [1], with *P. falciparum* and *P. vivax* being the primary drivers of morbidity and mortality [2]. The 2024 WHO malaria report indicates 263 million global cases in 2023, 11 million more than in 2022 [3]. While China has seen no major domestic outbreaks recently, overseas importation risks persist, challenging malaria prevention, diagnosis, and treatment.

Artemisinin resistance in *P. falciparum* further hinders control efforts [4]. *P*lasmodium** infection often induces splenomegaly, as the spleen is the key organ for processing infected red blood cells, and this process may activate splenic T cells to combat the parasite [5].

CD8 ⁺ T cells are critical in immune defense. Upon activation, they differentiate into cytotoxic T lymphocytes (CTLs) that target and eliminate infected cells [6]. CTLs limit pathogenic infections by directly destroying infected cells (*via* perforin/granzyme or Fas/FasL pathways) or releasing cytokines like IFN-γ and TNF-α to enhance immune responses against viral, bacterial, and parasitic infections [7]. During malarial liver infection, interferon signaling may impair Th1 and CD8 ⁺ T cell responses, leading to exhaustion of liver-resident CD8 ⁺ T cells [8]. Effector memory CD8 ⁺ T cells rapidly migrate to the liver within 6 hours of *Plasmodium* or bacterial infection to clear pathogens, a process accompanied by rapid upregulation of inflammatory genes, dependent on intrinsic LFA-1 expression in CD8 ⁺ T cells and hepatic phagocytes [9].

In chronic infections, prolonged exposure to antigens or inflammatory signals impairs CD8 ⁺ T cell function, a state termed “exhaustion”, characterized by reduced effector activity, elevated inhibitory receptors, metabolic dysfunction, impaired memory recall, and altered homeostatic proliferation [10]. LAG3, a co-inhibitory molecule, suppresses CD8 ⁺ T cell function by binding ligands [11]. During Plasmodium infection, LAG3 is detectable in blood, spleen, brain, and liver [12], making it a promising immunotherapeutic target with over 20 clinical trials underway. Studies show LAG3 signaling negatively regulates Th1 cell activation, inhibiting proliferation and cytokine secretion [13,14]. In PD-L1 knockout mice, anti-LAG3 monoclonal antibodies accelerate the clearance of blood-stage parasites [15].

Our previous research demonstrated that the transcription factor NFATc1 regulates PD1 expression [16]. NFATc, a key transcription factor in activated T cells regulated by calmodulin signaling, participates in bone remodeling and atherosclerotic calcification [17]. As a member of the NFATc family, NFATc1 is involved in B and T lymphocyte differentiation, heart valve formation, and bone remodeling [18–20]. NFATc1 deficiency impairs glycolysis in CD8 ⁺ T cells, highlighting its role in activating and regulating CD8 ⁺ T cell function.

This study investigates the phenotypic and functional characteristics of LAG3 ⁺ CD8 ⁺ T cells in the spleen of *Plasmodium yoelii* NSM-infected mice and explores the molecular mechanisms underlying LAG3 expression.

## Materials and methods

### Ethical statement

All animal protocols were approved by the Institutional Animal Care and Use Committee of Guangzhou Medical University (Ethics No. GY2020–134) and conducted following the committee’s guidelines to minimize suffering.

### Experimental animals and *Plasmodium* infection

Female C57BL/6 mice (6–8 weeks old) were purchased from the Animal Center of Guangzhou University of Chinese Medicine and housed in the SPF-level Animal Experiment Center of Guangzhou Medical University. Mice were randomly assigned to uninfected or infected groups. Infected mice received an intraperitoneal injection of 200 μL of a suspension containing 5 × 10⁶ infected red blood cells/mL (prepared by diluting peripheral blood from resuscitated infected mice with sterile PBS).

### Preparation of splenic lymphocyte suspensions

Mice were sacrificed, and spleens were harvested. After adding D-Hank’s solution, spleens were mechanically ground, and the homogenate was centrifuged. Red blood cell lysis solution was added, incubated for 5 minutes, and centrifuged. The pellet was resuspended in 5 mL complete medium for cell counting.

### RNA Preparation for Real-Time PCR

Cells were cryopreserved in Trizol (Invitrogen, USA) for RNA extraction. Quantitative PCR (qPCR) was performed using a CFX96 System (Bio-Rad, USA). Details of siRNAs and primers are provided in S1 Table.

### Single-cell sequencing

Spleens from uninfected and infected mice were processed into single-cell suspensions. Immune cells were labeled with CD45 flow cytometry antibodies, and samples were sent to Guangdong Yuanxin Biotechnology Co., Ltd. for single-cell sequencing. Data were analyzed using Loupe Browser software.

### Flow cytometry analysis

F or surface marker detection, single lymphocytes were stained with fluorescently labeled antibodies against mouse CD45, CD3, CD8, LAG3, CD4, CD19, NK1.1, γδTCR, PD1, TIM3, TIGIT, CD69, ICOS, CD25, CD62L, CD44, CD38, and Fixable Viability Dye (BioLegend) at 4°C for 30 minutes. For cytokine detection, lymphocytes were stimulated with phorbol myristate acetate (PMA, 20 ng/mL, Sigma, Germany) and ionomycin (1 mg/mL, Sigma) at 37°C for 4.5 hours, with brefeldin A (BFA, 10 mg/mL, Sigma, Germany) added after 1 hour. Cells were stained for surface markers, fixed/permeabilized using a Fixation/Permeabilization Solution Kit (555028, BD Biosciences) at 4°C for 20 minutes in the dark, then stained for IFN-γ and CD107a for 30 minutes. For NFATc1 and Ki67 detection, cells were treated with a transcription factor staining kit (00-5523-00, Invitrogen, USA) and stained with fluorescent antibodies for 30 minutes. Stained cells were analyzed using flow cytometry with CytExpert 1.1 software (Beckman Coulter, USA).

### Cell culture

Splenic lymphocytes were aseptically collected and seeded in 24-well plates (2 × 10^6^/mL), and stimulated with lysates from different numbers of uninfected RBC (uRBC), or iRBC plus anti-mouse CD28 monoclonal antibodies (1 μg/mL each). Cells were cultured at 37°C with 5% CO₂ for 48 hours.

### Magnetic Bead Sorting of CD8 ⁺ T Cells

Splenic lymphocytes were incubated with anti-mouse CD8a antibody-conjugated magnetic beads (Naive CD8a ⁺ T Cell Isolation Kit, Miltenyi Biotec) at 4°C for 30 minutes. Cells were loaded onto pre-rinsed columns in a magnetic field, and target cells were eluted after removing the magnet. Purity was verified by flow cytometry.

### Dual-luciferase reporter assay

HEK 293T cells were transfected with target plasmids using Lipofectamine 3000 (Invitrogen). Luminescence was measured using the Dual-Luciferase Reporter Assay System (Promega), with results calculated as the ratio of firefly to Renilla luciferase activity.

### siRNA transfection

HuT78 cells (60%–80% confluent) were transfected with siRNA using PEI 40000 (Yeasen) and cultured at 37°C with 5% CO₂ for 5 hours. Transfection efficiency was confirmed via fluorescence microscopy. Medium was replaced, and cells were cultured for 43 hours before RT-qPCR analysis.

### Statistical analysis

Data were analyzed using GraphPad Prism 9.0.0. Normally distributed data were compared using independent t-tests; multiple groups were analyzed by one-way ANOVA with LSD post-hoc tests. Non-normally distributed or heteroscedastic data were analyzed using the Mann-Whitney U test. Significance was defined as *p < 0.05 (**p < 0.01, ***p < 0.001, ****p < 0.0001), *ns* as *p* > 0.05.

## Results

**1. Plasmodium infection induces significant changes in splenic CD8**^**+**^ **T cells**

**Fig 1:**
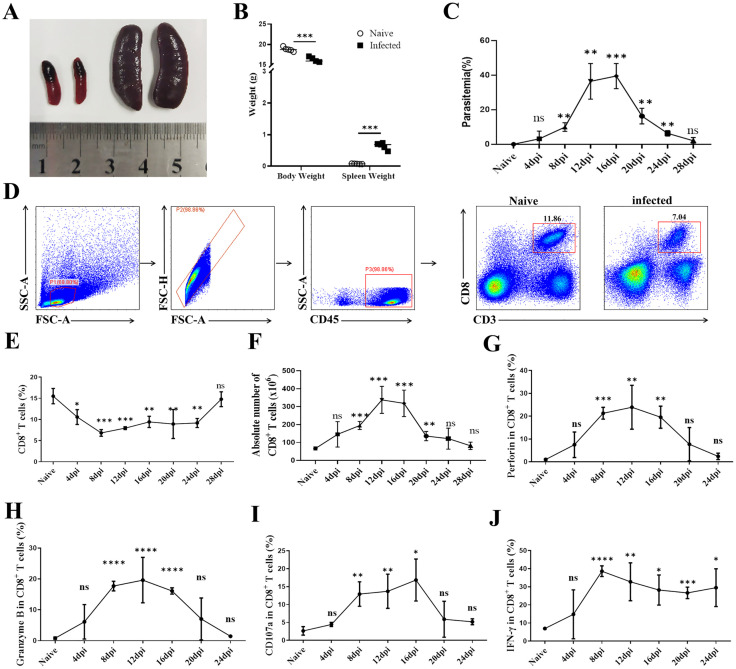
Changes in spleen CD8 + T cell content and function in Plasmodium-infected mice. **(A)** Representative spleen images of uninfected and infected mice. **(B)** Statistical analysis of mouse body weight and spleen weight. **(C)** Parasitemia was counted via tail vein blood smears under a microscope every 4 days post-Plasmodium infection (DPI). **(D-F)** Dynamic changes in the proportion and number of splenic CD8^+^ T cells detected by flow cytometry after infection. **(G-K)** Dynamic detection of Perforin, Granzyme B, CD107a, and IFN-γ secretion by splenic CD8^+^ T cells using flow cytometry. Each group included 4-6 mice, and experiments were repeated twice. *p < 0.05, **p < 0.01, ***p < 0.001, ****p < 0.0001, *ns*: *p* > 0.05.

To investigate the impact of *Plasmodium yoelii* on rodents, we initially measured the weights of infected and uninfected mice. Upon sacrifice, spleens were collected for photography and weighing (Fig 1A and 1B). Results showed that Plasmodium infection led to a significant decrease in mouse weight and notable changes in spleen characteristics, including increased volume and weight, and darkening of color. These observations suggest the accumulation of Plasmodium deposits on the spleen surface, causing severe organ damage and overall physiological harm. After infecting mice with Plasmodium, tail vein blood smears were prepared every 4 days to count the parasite rate (Fig 1C), revealing that the infection peaked between 12 and 16 days post-infection.

Current research has demonstrated the crucial role of CD8^+^ T cells in combating inflammation. We therefore investigated the impact of Plasmodium infection on the expression of splenic CD8^+^ T cells. A cohort of infected mice was euthanized every 4 days; spleen single-cell suspensions were isolated, and flow cytometry was used to monitor dynamic changes in the proportion and quantity of splenic CD8^+^ T cells (Fig 1D–1F). We also assessed changes in the ability of splenic CD8^+^ T cells to secrete cytotoxic functional molecules, such as Perforin, Granzyme B, and CD107a (Lysosomal associated membrane protein 1), as well as the cytokine IFN-γ (Fig 1G–1J). Our findings showed that as Plasmodium infection progressed, although the proportion of CD8^+^ T cells decreased post-infection, the absolute number of splenic CD8^+^ T cells increased significantly, peaking at 12–16 days post-infection. Concurrently, the secretion of toxic functional molecules and cytokines by CD8^+^ T cells followed a similar trend. Thus, the period 12–16 days post-infection is not only the most critical phase of infection in mice but also when the quantity and function of splenic CD8^+^ T cells undergo the most substantial changes. We therefore selected this time frame for subsequent experiments on CD8^+^ T cells.

To explore the role of splenic CD8^+^ T cells in combating Plasmodium infection, splenic immune cells (CD45^+^ cells) were isolated from uninfected and infected mice using flow cell sorting technology, followed by single-cell sequencing to analyze differential gene expression in splenic CD8^+^ T cells between the two groups (S2A and S2B Fig). Compared with uninfected mice, splenic CD8^+^ T cells from infected mice showed significant alterations in gene expression profiles, with elevated expression of cytotoxicity-related genes such as Gzma, Gzmb, Gzmk, and Prf1, as well as increased expression of the immunosuppressive molecule LAG3. Notably, LAG3 was absent in uninfected CD8^+^ T cells but highly expressed post-infection, suggesting a potential role in the infection process. Conversely, transcription factors Lef1 and Tcf7, which are associated with T cell development, were expressed at low levels in infected CD8^+^ T cells.

To further investigate the functions of these differentially expressed genes in splenic CD8^+^ T cells, we performed KEGG analysis (S1C Fig), GO analysis (S1D Fig), and GSEA analysis (S1E Fig) on CD8 + T cells from uninfected and infected mice. KEGG analysis showed that differentially expressed genes in CD8^+^ T cells from infected mice were enriched in various signaling pathways, particularly “Oxidative phosphorylation”. GO analysis (encompassing biological process [BP], cellular component [CC], and molecular function [MF]) revealed that differentially expressed genes in splenic CD8^+^ T cells from infected mice were associated with “Lymphocyte differentiation” and “generation of precursors” in BP, “metabolites and energy” in CC, and “Mitochondrial protein-containing complex” and “ribosome” in MF. GSEA analysis showed that differentially expressed genes in splenic CD8^+^ T cells from infected mice were enriched in pathways such as “Oxidative phosphorylation” and “Leukocyte transendothelial migration”. These findings suggest a strong correlation between splenic CD8^+^ T cells in infected mice and lymphocyte development.

**2. Plasmodium infection enhances LAG3 expression on splenic CD8**^**+**^ **T cells.**

**Fig 2:**
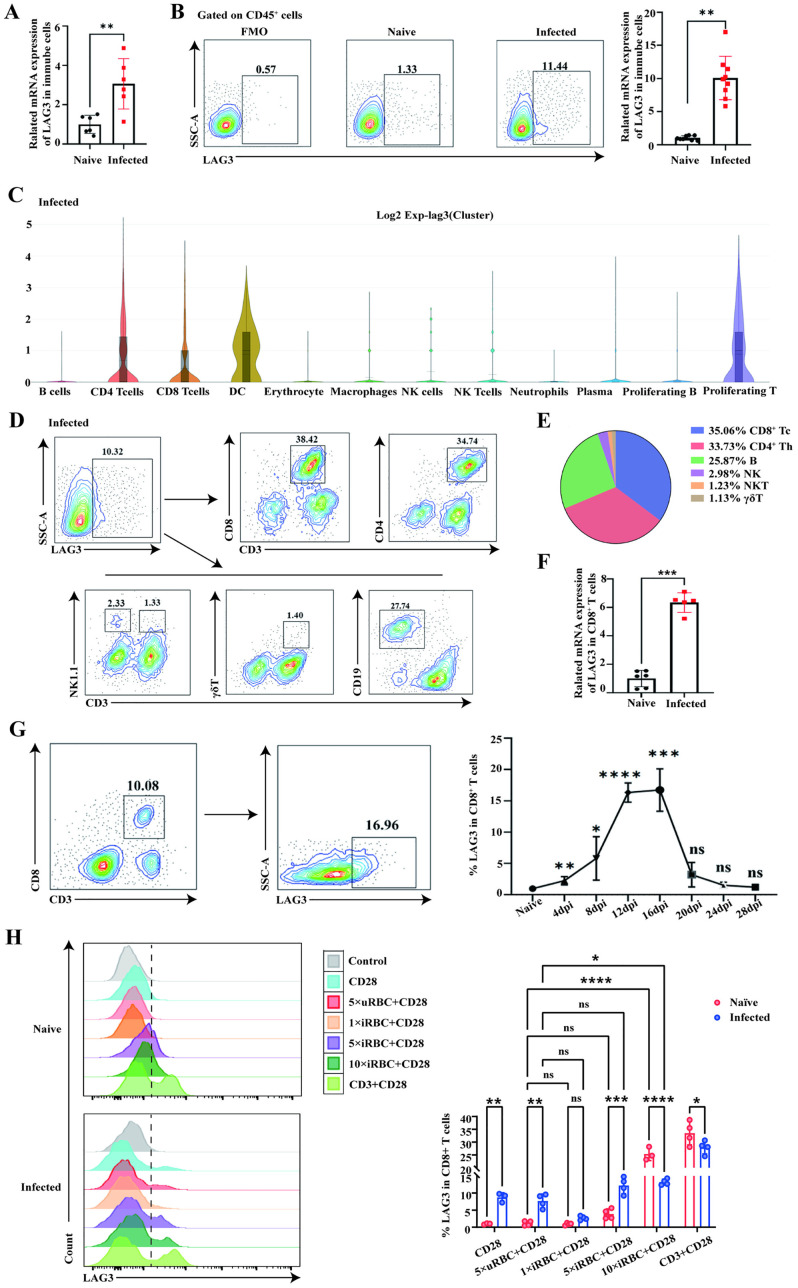
Plasmodium infection induces splenic LAG3^+^CD8^+^ T cells in mice. **(A)** RT-qPCR analysis of LAG3 mRNA levels in splenic immune cells from uninfected and infected mice. **(B)** Flow cytometric detection of LAG3 protein levels in splenic immune cells. **(C)** Violin plots showing LAG3 fluorescence intensity in splenic immune cells after Plasmodium infection. **(D, E)** Flow cytometry analysis of LAG3 distribution in splenic immune cells post-infection. **(F)** LAG3 mRNA levels in splenic CD8^+^ T cells (sorted by magnetic beads) from uninfected and infected mice, detected by RT-qPCR. **(G)** Dynamic changes in LAG3 protein expression in CD8^+^ T cells (detected by flow cytometry) in infected mice sacrificed every 4 days, with uninfected mice as controls. **(H)** spleen lymphocytes were isolated from uninfected mice and stimulated with lysates from 5-fold numbers of uRBC (5 × uRBC), 1-fold numbers of iRBC (1 × iRBC), 5-fold numbers of iRBC (5 × iRBC), or 10-fold numbers of iRBC (10 × iRBC), respectively, Anti-mouse CD28 monoclonal antibodies were plused, and LAG3 expression in CD8^+^ T cells was assessed by flow cytometry, 48 hours later. Each group included 4-6 mice, and experiments were repeated twice. *p < 0.05, **p < 0.01, ***p < 0.001, ****p < 0.0001, *ns*: *p* > 0.05.

Differential gene analysis of CD8^+^ T cells following Plasmodium infection revealed that lymphocyte activation gene 3 (LAG3) was absent in uninfected CD8^+^ T cells, but highly expressed post-infection (S2A and S2B Fig), indicating a potentially significant role of LAG3 in the infection response. To investigate LAG3 expression in immune cells following Plasmodium infection, RT-qPCR was used to detect LAG3 mRNA expression in spleen immune cells before and after infection. Results showed that LAG3 mRNA expression in splenocytes was significantly higher in infected mice than in uninfected mice (Fig 2A). Flow cytometry was then used to assess LAG3 protein expression pre- and post-infection (Fig 2B), revealing a notable elevation in LAG3 protein levels in spleen immune cells of infected mice compared to uninfected counterparts. Collectively, these results indicate a substantial upregulation of the inhibitory receptor LAG3 in spleen immune cells following Plasmodium infection.

To further clarify the distribution of LAG3 in immune cells, we analyzed its expression in different immune cell groups following Plasmodium infection. Using single-cell sequencing, we assessed the fluorescence intensity of LAG3 in various immune cell populations in the spleen of infected mice (Fig 2C), finding predominant expression of LAG3 on CD8^+^ T cells, CD4^+^ T cells, DC cells, and proliferating T cells post-infection. Flow cytometry was then used to identify LAG3^+^ cells in different immune cell subsets in mouse spleens following infection (Fig 2D and 2E). Results showed the hierarchy of LAG3^+^ immune cells in the spleen post-infection as follows: CD8^+^ T cells (CD3^+^CD8^+^), CD4^+^ T cells (CD3^+^CD4^+^), B cells (CD3^-^CD19^+^), NK cells (CD3^-^NK1.1^+^), NK T cells (CD3^+^NK1.1^+^), and γδT cells (CD3^+^γδT^+^). These results indicate that LAG3 expression is primarily concentrated in CD8^+^ T cells and CD4^+^ T cells in the spleen following Plasmodium infection.

To investigate LAG3 expression in splenic CD8^+^ T cells following Plasmodium infection, splenic CD8^+^ T cells were isolated from uninfected and infected mice using magnetic beads, and RT-qPCR was used to detect LAG3 levels. Results showed a significant upregulation of LAG3 mRNA in splenic CD8^+^ T cells post-infection compared to uninfected mice (*P* < 0.05, Fig 2F). Using uninfected mice as controls, we sacrificed infected mice every 4 days and monitored LAG3 protein expression in CD8^+^ T cells using flow cytometry. LAG3 protein expression in splenic CD8^+^ T cells followed a similar trend to the parasite rate, peaking at 12–16 days post-infection before declining to levels comparable to the uninfected group (Fig 2G). These findings suggest that infection leads to a notable increase in LAG3^+^CD8^+^ T cells in the spleens of mice.

To investigate LAG3 expression in CD8^+^ T cells following antigen stimulation, spleen lymphocytes were isolated from uninfected mice and stimulated with lysates from 5-fold numbers of uRBC (5 × uRBC), 1-fold numbers of iRBC (1 × iRBC), 5-fold numbers of iRBC (5 × iRBC), or 10-fold numbers of iRBC (10 × iRBC), respectively. Anti-mouse CD28 monoclonal antibodies were plused, and LAG3 expression in CD8^+^ T cells was assessed by flow cytometry, 48 hours later (Fig 2H). Results showed that although1 × iRBC could not induce higher percentages of LAG3^+^CD8^+^ T cells compared to 5 × uRBC, 5 × iRBC could induce higher percentages of LAG3^+^CD8^+^ T cells in splenocytes from infected mice (*P* < 0.05). While 10 × iRBC could induce higher percentages of LAG3^+^CD8^+^ T in splenocytes from both normal and infected mice (*P* < 0.05), the percentage of LAG3^+^CD8^+^ T induced in splenocytes from normal mice was higher than that induced in splenocytes from infected mice (*P* < 0.05).

**3. Plasmodium infection causes LAG3**^**+**^**CD8**^**+**^ **T cells to exhibit characteristics of activation, high efficacy, and high proliferation.**

**Fig 3:**
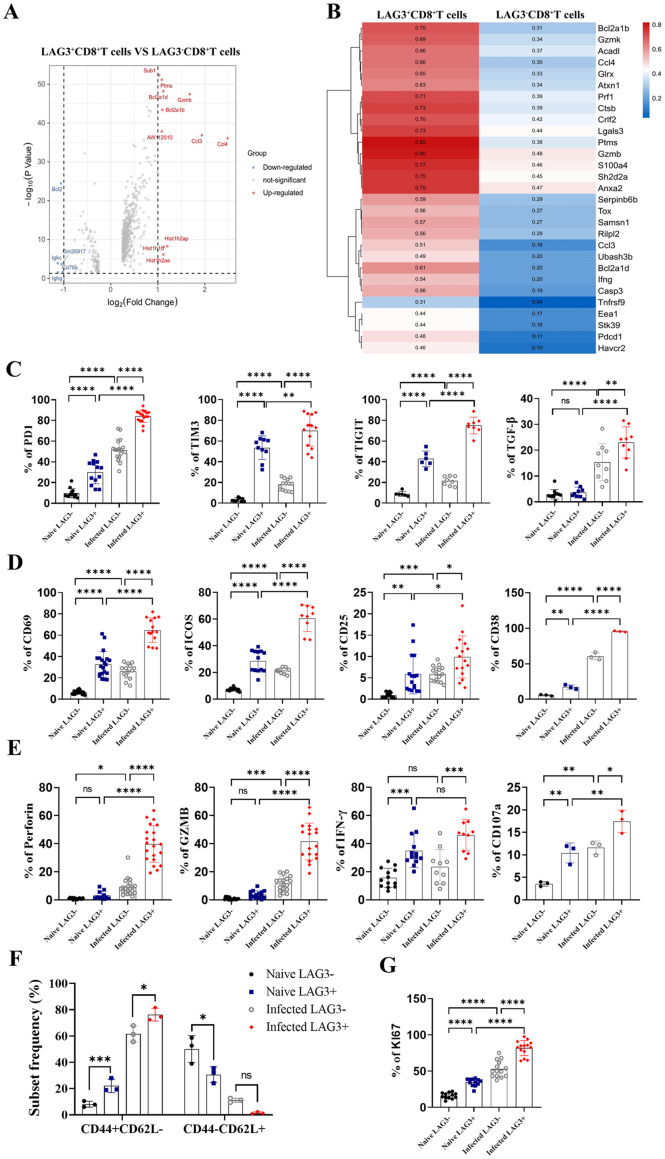
Phenotypic and functional characteristics of splenic LAG3^+^CD8^+^ T cells in Plasmodium-infected C57BL/6 mice. **(A, B)** Differential gene analysis of splenic LAG3^+^CD8^+^ T cells vs. LAG3^-^CD8^+^ T cells in infected mice using single-cell sequencing data, visualized by a volcano plot **(A)** and heatmap **(B)**. **(C)** Flow cytometric detection of co-expression of LAG3 and inhibitory molecules (PD1, TIM3, TIGIT, TGF-β) in CD8^+^ T cells. **(D)** Flow cytometry analysis of co-expression of LAG3 and activation markers (CD69, ICOS, CD25, CD38) in CD8 + T cells from uninfected and infected mice. **(E)** Flow cytometric detection of Perforin, Granzyme B, IFN-γ, and CD107a secretion in LAG3^+^CD8^+^ T cells from uninfected and infected mice. **(F)** Effector and resting phenotypes of splenic LAG3^+^CD8^+^ T cells vs. LAG3^-^CD8^+^ T cells detected by flow cytometry. **(G)** Flow cytometric analysis of co-expression of LAG3^+^CD8^+^ T cells and proliferation marker Ki67 before and after infection. Each group included 4-6 mice, and experiments were repeated three times. *p < 0.05, **p < 0.01, ***p < 0.001, ****p < 0.0001, *ns*: *p* > 0.05.

To investigate the relationship between LAG3 and other molecules in CD8^+^ T cells during Plasmodium infection, single-cell sequencing data were used to compare gene expression between LAG3^-^CD8^+^ T cells and LAG3^+^CD8^+^ T cells in the spleen of infected mice (Fig 3A and 3B). Analysis revealed that, compared to LAG3^-^CD8^+^ T cells, the LAG3^+^CD8^+^ T cell population in the spleen of infected mice showed increased co-expression of immunosuppressive molecules Pdcd1 (PD1) and Havcr2 (TIM3), as well as functional factors Prf1 (Perforin), Gzmb (Granzyme B), Gzmk (Granzyme K), and the inflammatory cytokine Ifng (IFN-γ).

Recent studies have highlighted the significant impact of exhaustion molecules on CD8^+^ T cell function [21]. LAG3 is primarily expressed in activated T cells, NK cells, and DC cells [22]. PD1 is expressed in various immune cells, including B cells, T cells, NK cells, and macrophages [23]. TIGIT, which has Ig and ITIM domains, is highly expressed in activated T cells, and TIM3, originally expressed on Th1 cells, has recently been found to play an important role in the connection between CD8^+^ T cells and antiviral infection [24]. Blocking exhaustion molecular receptors has been shown to restore the activation, proliferation, and killing capabilities of exhausted CD8^+^ T cells [25], and the expression and function of exhaustion molecules in CD8^+^ T cells are closely related to TGF-β [26]. To further investigate the correlation between splenic LAG3^+^CD8^+^ T cells and immunosuppressive molecules, flow cytometry was used to detect the expression of LAG3, PD1, TIM3, TIGIT, and TGF-β in splenic CD8^+^ T cells (S2A and S3C Figs). Results showed that PD1, TIM3, TIGIT, and TGF-β expression in CD8^+^ T cells was higher in infected mice than in uninfected mice. Notably, LAG3^+^CD8^+^ T cells exhibited higher expression of these inhibitory molecules than LAG3^-^CD8^+^ T cells under both uninfected and infected conditions, and the proportion of co-inhibitory molecules in LAG3^+^CD8^+^ T cells increased significantly following infection. These findings suggest that Plasmodium infection induces the co-expression of LAG3 and immunosuppressive molecules in CD8 + T cells.

The cell surface glycoprotein CD69 is crucial in the early activation of T cells, with low expression in the resting state and significant upregulation upon T cell activation [27]. Inducible T cell co-stimulator (ICOS) is involved in activated T cells, contributing to processes such as cell proliferation and activation, particularly in regulating CD8 + T cell and B cell signaling [28]. CD25, also known as IL-2 receptor α chain, is widely present in regulatory and resting memory T cells, playing a pivotal role in IL-2 production and T cell activation [29]. CD38, a membrane protein, serves as an important marker of activated T cells [30]. To further investigate the phenotypic characteristics of LAG3^+^CD8^+^ T cells following Plasmodium infection, single-cell suspensions were prepared from spleen cells of uninfected and infected mice, and the expression of activation molecules CD69, ICOS, CD25, and CD38 was analyzed (S2B and S3D Figs). Results showed that activating factors were significantly more abundant in LAG3^+^CD8^+^ T cells than in LAG3^-^CD8^+^ T cells, regardless of infection status, and LAG3^+^CD8^+^ T cell activation was notably higher post-infection than in uninfected LAG3^+^CD8^+^ T cells. These results indicate that LAG3^+^CD8^+^ T cells exhibit heightened activation following Plasmodium infection.

Single-cell sequencing data indicated that this cell subset had high expression levels of cytotoxic molecules such as Gzmk, Gzmb, Prf1, and the inflammatory cytokine IFN-γ (S1A and S1B Fig). Cytotoxic CD8 + T cells (CTLs) are crucial in eliminating infected red blood cells through the release of cytotoxic granules and cytokine secretion. To further elucidate the cytokine secretion capacity of LAG3^+^CD8^+^ T cells, flow cytometry was used to monitor changes in the protein levels of Perforin, Gzmb, IFN-γ, and CD107a (Figs 3E and S2C). Findings showed that post-infection, LAG3^+^CD8^+^ T cells had a significantly enhanced ability to secrete cytokines compared to both uninfected LAG3^+^CD8^+^ T cells and infected LAG3^-^CD8^+^ T cells.

CD62L, typically found on naive T cells (and also present on the surface of B cells), is crucial for the migration and recruitment of naive T cells, and its expression is inhibited by protease secretion under various stimulation conditions33. To further investigate phenotypic characteristics, CD8^+^ T cells were categorized into resting phenotype (CD44^-^CD62L^+^) and effector phenotype (CD44^+^CD62L^-^) based on CD44 and CD62L expression levels. Flow cytometry was used to assess the phenotypic differentiation of LAG3^-^CD8^+^ T cells and LAG3^+^CD8^+^ T cells in the spleens of uninfected and infected mice (Figs 3F and S2D). Results showed that, regardless of infection status, LAG3^+^CD8^+^ T cells had a higher proportion of effector phenotypes than LAG3^-^CD8^+^ T cells. Additionally, in the normal state, the resting phenotype proportion of LAG3^-^CD8^+^ T cells was notably greater than that of LAG3^+^CD8^+^ T cells, suggesting that following Plasmodium infection, LAG3^-^CD8^+^ T cells are in a highly activated state that may impact inflammatory responses in the body.

Cell proliferation levels indicate both the extent of cell activation and the cell’s responsiveness to stimulation. Flow cytometry was therefore used to assess the expression of the proliferation marker Ki67 in splenic LAG3^+^CD8^+^ T cells (Figs 3G and S2D). Results showed that the proliferation capacity of LAG3^+^CD8^+^ T cells was notably greater than that of LAG3^-^CD8^+^ T cells under both uninfected and infected conditions, and LAG3^+^CD8^+^ T cells in the spleens of infected mice exhibited enhanced proliferation ability compared to those in uninfected mice. These outcomes indicate that following Plasmodium infection, splenic LAG3^+^CD8^+^ T cells in mice are highly activated and proliferative.

**4. NFATc1 binds to the LAG3 promoter and regulates its expression.**

To further elucidate the functional changes of LAG3^+^CD8^+^ T cells, we analyzed single-cell transcription sequencing data. Post-infection, splenic CD8^+^ T cells were categorized into LAG3^-^CD8^+^ T cells and LAG3^+^CD8^+^ T cells, and differential gene analysis was performed. The identified differentially expressed gene sets underwent functional enrichment analysis (S3A–S3C Fig). GO analysis (encompassing BP, CC, and MF) showed that differentially expressed genes in splenic LAG3^+^CD8^+^ T cells from infected mice were enriched in “Lymphocyte differentiation” and “Regulation of T cell activation” in BP; “Organelle inner membrane” and “Mitochondrial inner membrane” in CC; and “Ubiquitin-like protein ligase binding” and “Ubiquitin protein ligase binding” in MF (S3A Fig). KEGG analysis revealed enrichment in the “T cell receptor signaling pathway” and “PD-L1 expression and PD1 checkpoint pathway in cancer” (S3B Fig), and GSEA analysis showed enrichment in pathways such as “T cell receptor signaling pathway”, “PD-L1 expression and PD1 checkpoint pathway in cancer”, and “Cytokine-cytokine receptor interaction” (S3C Fig). These findings indicate that Plasmodium infection enhances cytotoxicity mediated by LAG3^+^CD8^+^ T cells, highlighting their crucial regulatory role in T cell proliferation and activation.

**Fig 4:**
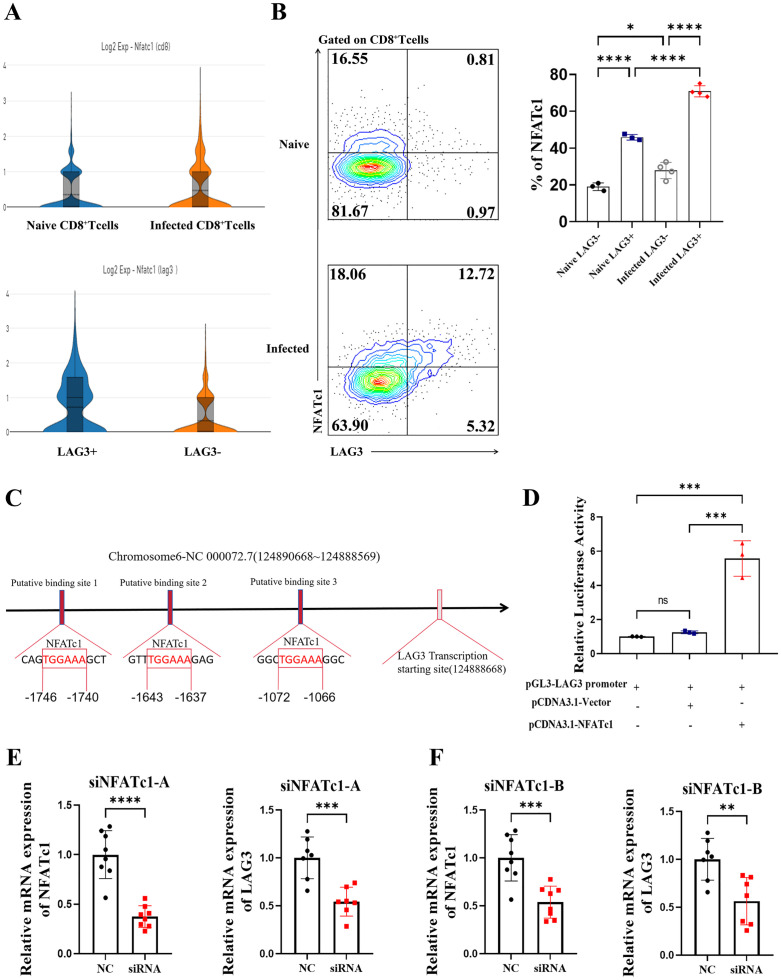
NFATc1 binds to the LAG3 promoter sequence and regulates its expression. **(A)** Violin plots showing NFATc1 expression in CD8^+^ T cells under uninfected vs. infected conditions, in splenic CD8^+^ T cells at 12 days post-infection, and in LAG3^+^CD8^+^ T cells *vs*. LAG3-CD8^+^ T cells. **(B)** Flow cytometric detection of co-expression of LAG3 and NFATc1 on CD8^+^ T cells. **(C)** Predicted binding sites of transcription factor NFATc1 in the LAG3 promoter sequence via the JASPAR database. **(D)** Relative fluorescence intensity detected by dual-luciferase reporter assay in HEK 293T cells co-transfected with pGL3-LAG3 promoter plasmid and either pCDNA3.1(+)-NFATc1 plasmid or pCDNA3.1(+)-Vector plasmid for 48 hours. **(E, F)** qPCR detection of NFATc1 and LAG3 mRNA levels in HuT78 cells 48 hours after transfection with NFATc1-targeting siRNA. Each group included 4-6 mice, and experiments were repeated three times. *p < 0.05, **p < 0.01, ***p < 0.001, ****p < 0.0001, *ns*: *p* > 0.05.

LAG3^+^CD8^+^ T cells exhibit characteristics of activation, high efficacy, and high proliferation under both normal and infected conditions, underscoring the importance of investigating the regulatory mechanisms of LAG3 expression. Our initial findings indicate that NFATc1 can trigger PD1 expression in the spleens of Plasmodium-infected mice16, and LAG3 and PD1 are closely interconnected in the immune response pathway. Whether NFATc1 affects the expression of LAG3 (an immunosuppressive molecule similar to PD1) remains to be studied. To elucidate the differential expression of NFATc1 in LAG3^+^CD8^+^ T cells versus LAG3^-^CD8^+^ T cells, single-cell sequencing was used to analyze NFATc1 expression in CD8 + T cells under normal and infected conditions (Fig 4A). Results showed that NFATc1 expression in splenic CD8^+^ T cells was significantly upregulated in mice infected for 12 days compared to uninfected mice.

Moreover, in splenic CD8^+^ T cells of mice infected for 12 days, the average fluorescence intensity of NFATc1 in LAG3^+^CD8^+^ T cells was notably higher than in LAG3^-^CD8^+^ T cells. Flow cytometry analysis revealed significantly higher NFATc1 expression in LAG3^+^CD8^+^ T cells than in LAG3^-^CD8^+^ T cells regardless of infection status, and NFATc1 protein levels in LAG3^+^CD8^+^ T cells were notably higher in infected mice than in uninfected mice (Fig 4B). These findings indicate a strong association between NFATc1 expression and LAG3^+^CD8^+^ T cells, with a more pronounced increase observed post-Plasmodium infection.

To investigate the potential role of the transcription factor NFATc1 in regulating LAG3 expression, the promoter sequence of the mouse LAG3 gene (encoding CD233) was queried using NCBI, and the JASPAR database was used to predict NFATc1 binding sites on the LAG3 promoter sequence. Three binding sites were identified, located at -1746 bp to -1740 bp, -1643 bp to -1637 bp, and -1072 bp to -1066 bp upstream of the transcription start site (Fig 4C). Based on these predictions, the LAG3 gene promoter sequence and NFATc1 gene coding sequence were retrieved from NCBI, and the pGL3-LAG3 promoter plasmid and pCDNA3.1(+)-NFATc1 plasmid were synthesized. pRL-TK was introduced as an internal control plasmid, along with the pGL3-LAG3 promoter plasmid and either the pCDNA3.1(+)-empty vector plasmid or pCDNA3.1(+)-NFATc1 plasmid for co-transfection. After 48 hours of co-transfection, a dual-luciferase reporter gene assay was used to investigate the regulatory binding of NFATc1 to the LAG3 promoter sequence (Fig 4D). Results showed that relative firefly luciferase activity was significantly higher in cells co-transfected with the pCDNA3.1(+)-NFATc1 plasmid than in those co-transfected with the pCDNA3.1(+)-Vector plasmid, suggesting that NFATc1 can bind to the LAG3 promoter sequence and enhance its transcriptional expression.

To further confirm NFATc1’s ability to bind to the LAG3 gene promoter sequence, small interfering RNA (siRNA) targeting two different sites of NFATc1 was introduced into HuT78 cells to disrupt NFATc1 expression, and mRNA changes were assessed using RT-qPCR (Fig 4E and 4F). Results showed that siRNA effectively reduced NFATc1 mRNA levels in HuT78 cells, leading to a significant decrease in LAG3 mRNA levels. These results support the notion that NFATc1 binds to the LAG3 gene promoter sequence, thereby influencing its expression and the transcriptional activity of downstream genes.

## Discussion

During the intraerythroid phase, the spleen, being the largest lymphoid organ, assumes a pivotal role in triggering the immune response and safeguarding the host against Plasmodium [31]. Research has clearly shown that transfusing radiolabeled autologous red blood cells during acute Plasmodium infection substantially heightens the spleen’s efficiency in clearing red blood cells [32]. Splenic congestion stands as a primary culprit for splenomegaly in malaria patients and might contribute to the development of anemia. The accumulation of red blood cells in the spleen exacerbates splenomegaly and related symptoms [33]. Our results vividly demonstrate that upon infection with Plasmodium, the spleen, which is the largest lymphoid organ in mice, suffered damage, discoloration, enlargement, and a marked increase in weight. Simultaneously, the weight of the mice decreased significantly, hinting at the activation of the immune response post-infection. This implies that immune cells within the spleen likely play a crucial part in the infection process.

Research has indicated that following Plasmodium infection, CD8^+^ T cells continuously migrate and circulate within the body to identify infected red blood cells and subsequently eliminate these target cells through cytotoxicity [34]^.^ According to the presented findings, as the Plasmodium infection progresses, there is a conspicuous rise in the number of CD8^+^ T cells. Additionally, there is a gradual augmentation in the secretion of cytotoxic functional molecules such as Perforin, Granzyme B, CD107a, and the cytokine IFN-γ. This reveals a strong correlation between the immune function of CD8^+^ T cells and the severity of Plasmodium infection. Moreover, the single-cell sequencing results vividly show significant gene expression variances in splenic CD8^+^ T cells between uninfected and infected mice. These distinct genes are closely linked to pathways related to lymphocyte development, highlighting the pivotal role of CD8^+^ T cells in combating Plasmodium infection. By secreting cytotoxic functional molecules like Perforin, Granzyme B, CD107a, and cytokines such as IFN-γ, CD8^+^ T cells may enhance their effector function, thus exerting anti-Plasmodium infection effects.

LAG3 plays a crucial role in activating diverse immune cells, including T cells, regulatory T cells, NK cells, B cells, plasmacytoid dendritic cells, and neurons [35]. In malaria patients, there is a notable elevation in the expression levels of the inhibitory receptors TIM3 and LAG3 in T cells [36]. Our findings reveal that Plasmodium infection can upregulate LAG3 levels in immune cells of the mouse spleen, with predominant expression on T cells. This post-infection up-regulation of LAG3 implies its involvement in modulating immune responses and maintaining immune homeostasis. Employing flow cytometry, single-cell sequencing, and RT-qPCR, we observed alterations in LAG3 levels in CD8^+^ T cells before and after infection. Our results indicated a substantial increase in LAG3^+^CD8^+^ T cells in mouse spleens following Plasmodium infection. Additionally, dynamic monitoring disclosed a correlation between LAG3 levels in CD8^+^ T cells and parasitemia rates, reaching a peak at 12–16 days post-infection before declining. This temporal pattern suggests a close association between LAG3 expression and Plasmodium-induced inflammation. In the literature, it has been reported that LAG3 is mainly activated by TCR receptors or induced by cytokines such as IL-12, IL-27, IL-15, IL-2, and IL-7 [37]. Our study revealed a significant increase in LAG3 expression in CD8^+^ T cells in iRBC stimulated splenocytes from both normal and infected ice *in vitro*. Following infection, immunosuppressive molecules such as PD1, LAG3, TIM3, and TIGIT in CD8^+^ T cells were shown to regulate T cell function, leading to impaired proliferation, cytokine production, and target cell lysis [38]. Therefore, it is suggested that post-Plasmodium infection, the up-regulation of LAG3 in CD8^+^ T cells plays a crucial role in modulating effector function and influencing the immune response during Plasmodium infection.

LAG3^+^CD8^+^ T cells are indispensable in combating Plasmodium infection [36]. The activation status and characteristics of these cells are indicative of their anti-infection capabilities. Research shows that well-established markers like CD69, ICOS, CD25, and CD62L are commonly used to assess T cell activation [39–41]. Furthermore, activated LAG3^+^CD8^+^ T cells often co-express inhibitory receptors like PD1 and TI [36,42]. Our results demonstrated that LAG3^+^CD8^+^ T cells co-expressed inhibitory receptors such as PD1, TIM3, and TIGIT, along with activation indicators like CD69, ICOS, CD38, and CD25, and exhibited a significantly higher proliferation capacity compared to LAG3^-^CD8^+^ T cells. These findings suggest that LAG3^+^CD8^+^ T cells become highly activated post-infection and actively contribute to the immune response against Plasmodium.

NFATc1 is a key factor in activating and regulating CD8^+^ T cell function. Recent studies have increasingly focused on NFAT in CD8^+^ T cells, exploring aspects such as the nuclear translocation of NFATc1 post-chronic infection and NFATc1 expression in CD8^+^ T cells lacking effector capacity [43,44]. Our previous research identified NFATc1 as the primary transcription factor in PD-1^+^CD4^+^ T cells following Plasmodium infection [16]. In this study, single-cell sequencing results revealed an increase in NFATc1 expression in CD8^+^ T cells post-infection. Flow cytometry data indicated the coexpression of LAG3 and NFATc1 in CD8^+^ T cells, regardless of the infection status, suggesting NFATc1’s influence on LAG3 expression. This was predicted and confirmed using dual fluorescence reporters. Subsequent transfection of HuT78 cells with NFATc1-targeting siRNA led to a significant reduction in NFATc1 and LAG3 mRNA levels, corroborating the notion of NFATc1’s direct regulation of LAG3 expression. These findings demonstrate the binding of NFATc1 to the LAG3 promoter sequence and its regulatory impact on LAG3 expression.

In conclusion, our research indicates that Plasmodium infection can lead to an increase in the expression of LAG3 in CD8^+^ T cells, which exhibit heightened activation and functionality. NFATc1 can up-regulate LAG3 expression. However, after different Plasmodium infections, the characteristics (quantity, phenotype, function, localization) of LAG3^+^CD8^+^ T cells in the spleen may display significant differences. This difference is mainly driven by the biological characteristics of Plasmodium (antigen load, infection cycle, immune evasion strategies) and the remodeling of the host immune microenvironment. Further comparative studies will provide an important basis for malaria vaccine design (such as immune enhancement strategies targeting LAG3).

## Supporting information

S1 Table Detailed information of used siRNAs and primers. (DOCX)

S1 Fig Differential gene analysis of CD8^+^ T cells after Plasmodium infection. (A, B) Single-cell sequencing analysis of differential gene expression in splenic CD8^+^ T cells from uninfected vs. infected mice, visualized by volcano plot (A) and heatmap (B). (C-E) KEGG (C), GO (D), and GSEA (E) analyses of differential genes in CD8^+^ T cells from uninfected vs. infected mice based on single-cell sequencing data.
(TIF)

S2 Fig Gating strategy for flow cytometry. (TIF)

S3 Fig Differential gene enrichment analysis of LAG3^-^CD8^+^ T cells vs. LAG3^+^CD8^+^ T cells post-infection. (A-C) Functional enrichment analyses of differential genes in splenic LAG3^-^CD8^+^ T cells vs. LAG3^+^CD8^+^ T cells from infected mice, including GO (A), KEGG (B), and GSEA (C) analyses.
(TIF)

S4 Fig Proportions of different immune cell populations in the spleen of Plasmodium-infected mice. CD45^+^ cells were sorted from the spleens of normal and Plasmodium-infected mice (12 days post-infection) by FACS. Single-cell transcriptome sequencing was performed, and proportions of different immune cell populations are shown.
(TIF)
