# Supplementary material for: NFATc1 regulates LAG3+CD8+ T cells in the spleen of mice infected with Plasmodium yoelii NSM
Source: PLoS Negl Trop Dis. 2025 Oct 6;19(10):e0013605. doi: 10.1371/journal.pntd.0013605 (PMC12551951; doi:10.1371/journal.pntd.0013605)
Supplement: S1 Table — (DOCX) [file pntd.0013605.s001.docx]

**S1**_**Table: Detailed information for the used siRNAs and primers**

| **Gene** | **Forward Primer** | **Reversed Primer** |
| --- | --- | --- |
| β-actin (**mouse**) | CCGTAAAGACCTCTATGCCAA | GGGTGTAAAACGCAGCTCAGTA |
| LAG3 (**mouse**) | GAGTGGGGACCCCTTCTTTG | TTCCAGATGCCGGGGTTAC |
| β-actin (**human**) | CCTGGCACCCAGCACAAT | GGGCCGGACTCGTCATAC |
| LAG3 (**human**) | CCACCTCCTGCTGTTTCTCATCC | TCCCTGGCTCACCTGTCTTCTC |
| NFATc1 (**human**) | CAAGCCGAATTCTCTGGTGGT | ATGGCGTTACCGTTGGCG |
| **Gene** | **Forward Primer** | **Reversed Primer** |
| β-actin (**mouse**) | CCGTAAAGACCTCTATGCCAA | GGGTGTAAAACGCAGCTCAGTA |
